# Labor Induction with Synthetic Oxytocin and Infantile Colic: A Case–Control Study

**DOI:** 10.3390/medicina61111908

**Published:** 2025-10-24

**Authors:** Cristina Suárez-Fraga, Óscar Rodríguez-Nogueira, Arrate Pinto-Carral, Raquel Leirós-Rodríguez, María José Álvarez-Álvarez

**Affiliations:** 1 ECOM Physical Therapy and Osteopathy Clinic, Nursing and Physical Therapy Department, Universidad de León, 24004 Ponferrada, León, Spain; csuaf@unileon.es; 2 SALBIS Research Group, Nursing and Physical Therapy Department, Universidad de León, 24004 Ponferrada, León, Spain; orodn@unileon.es (Ó.R.-N.); apinc@unileon.es (A.P.-C.)

**Keywords:** infantile colic, synthetic oxytocin, epidural analgesia, head circumference, feeding, case-control study

## Abstract

*Background and Objectives*: Infantile colic affects 15–40% of infants ≤ 5 months, burdening families and health systems. While the effects of intrapartum oxytocin on neonatal outcomes have been widely investigated, its potential link with infantile colic remains poorly understood. We evaluated whether synthetic oxytocin is associated with infantile colic during the first five months of life and explored neonatal head circumference, feeding type and epidural anesthesia as additional factors. *Materials and Methods*: Prospective 1:1 matched case–control study in three Spanish pediatric outpatient clinics. Parents of 76 term infants aged 0–5 months (38 cases, 38 controls) completed face-to-face structured interviews documenting synthetic oxytocin and epidural use, infant anthropometry and feeding pattern. Infantile colic was diagnosed by Rome IV criteria. Associations were estimated with conditional logistic regression, producing adjusted odds ratios and 95% confidence intervals. *Results*: Synthetic oxytocin was used in 57.9% of deliveries and epidural anesthesia in 81.6%. Synthetic oxytocin showed no association with infantile colic (aOR 1.24; 95% CI 0.50–3.09). Epidural strongly predicted synthetic oxytocin exposure (aOR 4.55; 95% CI 1.28–16.20) but had no independent link to infantile colic. Infants with colic had a smaller mean head circumference at birth, although this difference did not remain significant after adjusting for gestational age, likely reflecting limited sample size. Synthetic oxytocin was not associated with breastfeeding status. *Conclusions*: In this cohort, intrapartum synthetic oxytocin was not related to infantile colic or to feeding difficulties. Smaller head circumference among colic cases may warrant further investigation as a potential risk marker. The high co-use of synthetic oxytocin and epidural underscores the need for larger longitudinal studies to clarify their peripartum–neonatal interactions.

## 1. Introduction

Infantile colic (infC) is marked by episodes of intense, hard-to-console crying. A classic definition from 1954 by Wessel and colleagues introduced the “rule of threes”: crying for more than 3 h per day, at least 3 days per week, for at least 3 weeks [1]. There is still no single, uniform definition; a recent review that incorporates the Rome IV Criteria continues to find heterogeneity in how colic is defined and measured, reflecting variability in diagnostic approaches [2]. Even the term “colic” has been questioned so some authors prefer “excessive infant crying” to emphasize a likely heterogeneous syndrome with multiple causes beyond a simple intestinal spasm.

Over time, efforts have aimed to standardize diagnosis. An international expert group on functional gastrointestinal disorders developed the symptom-based Rome Criteria, which are updated periodically. In the most recent revision (Rome IV), infC is defined as recurrent episodes of irritability, restlessness, or inconsolable crying of sudden onset and without apparent cause, lasting ≥ 3 h per day, occurring ≥ 3 days per week for at least 1 week, in an otherwise healthy infant under 5 months with appropriate growth [3].

Prevalence estimates vary with the definition and population studied. Broadly, colic affects about 15–20% of infants under five months; a recent global review using updated Rome IV definitions reports a prevalence of 10–15% [2]. Although colic is typically benign and self-limited (most cases resolve by 3–4 months), it causes substantial concern for families. It is linked to frequent pediatric visits in the first months and can reduce family quality of life due to the stress and anxiety associated with inconsolable crying [2].

Etiology is multifactorial, involving intertwined gastrointestinal, biological, and psychosocial factors. More recent metagenomic and functional work also reports distinct microbial profiles in infants with colic [4,5]. Together, these findings support a role for early intestinal colonization and the gut–brain axis, consistent with the reclassification of these conditions as disorders of gut–brain interaction [3].

Perinatal factors around birth may also play a role, though evidence remains limited. In particular, labor induction or augmentation with synthetic oxytocin (synOTC) has attracted attention given its widespread use and potential neonatal effects. SynOTC is among the most common interventions in modern obstetrics; its use for labor augmentation varies substantially across units and countries [6,7]. While exogenous oxytocin offers obstetric benefits, optimization of dosing and safety is ongoing [8,9].

Contemporary studies describe less efficient sucking patterns in the first 24–48 h and a lower likelihood of exclusive breastfeeding at one month after intrapartum oxytocin exposure [10]. Mechanistically, animal experiments suggest that maternally administered exogenous oxytocin can reach the fetal circulation, potentially influencing developing neonatal neurophysiology [10]. A possible link between oxytocin-induced labor and certain neurodevelopmental outcomes has also been discussed, although the epidemiology is mixed and vulnerable to confounding by indication [11].

In summary, while the effects of intrapartum oxytocin on neonatal and early neurodevelopmental outcomes have been widely investigated, its potential relationship with infantile colic remains largely unexplored. This raises a practical question: does oxytocin-based induction influence the occurrence of infC? Evidence remains scarce. Accordingly, the primary objective of this study was to examine whether intrapartum synOTC used to induce labor is related to the subsequent development of infC during the first five months of life. As secondary objectives, we explored associations with other perinatal and neonatal variables (e.g., feeding type, anthropometric characteristics) and quantified oxytocin use in our study population.

## 2. Materials and Methods

### 2.1. Study Design and Setting

We conducted an observational, analytical, 1:1 matched case–control study in pediatric outpatient settings, and the primary aim of the study was to assess whether intrapartum exposure to synthetic oxytocin is associated with the occurrence of infantile colic during the first five months of life. Recruitment took place from March to June 2022 across three private pediatric physiotherapy clinics in Ourense and Pontevedra (Galicia, Spain) and one public primary care center (Centro de Salud de La Palomera, León, Spain). Investigators used an interviewer-administered, structured questionnaire developed ad hoc and applied Rome IV criteria [3] to ascertain infC.

Matching was performed in a 1:1 ratio based on gestational age (±1 week). The matching process was carried out by the trained nurses and physiotherapists who conducted the parental interviews and data extraction. Although complete masking was not feasible, standardized procedures were applied to minimize bias: data were cross-checked against clinical records, and the final matched dataset was anonymized prior to statistical analysis.

### 2.2. Participants

Eligible participants were term infants aged 0–5 months at the time of interview.

The eligibility criteria were as follows: Inclusion criteria were infants aged between 15 days and 5 months whose parents or legal guardians had provided written informed consent to participate in the study. Exclusion criteria were infants diagnosed with gastrointestinal disorders (such as peritonitis, hepatitis, intestinal intussusception, ulcerative colitis, Crohn’s disease, etc.) and/or congenital pathologies (such as Down syndrome, Noonan syndrome, DiGeorge syndrome, cystic fibrosis, spina bifida, hypo- or hyperthyroidism, etc.).

For the definition of cases and controls, the following criteria were applied. Case: Any infant aged 0–5 months during the data collection period who met the Rome IV criteria for infantile colic, fulfilled all inclusion criteria, and did not meet any exclusion criteria. Control: Any infant aged 0–5 months during the data collection period who did not present infantile colic, fulfilled all inclusion criteria, and did not meet any exclusion criteria.

Enrollment followed a 1:1 matched design (cases–controls).

### 2.3. Sample Size and Sampling

The sample size (*n* = 62, 31 case–control pairs) was calculated following the guidelines of the Epidemiology Service of Hospital Juan Canalejo (A Coruña, Spain) aiming to detect an odds ratio of 2.0 with 95% confidence and 80% power. The calculation assumed an exposure frequency of 40% among cases and 10% among controls, based on a pilot analysis of patients attending Fisioterapia Ecom Clinic between 2014 and 2022. We ultimately enrolled 76 infants (38 case–control pairs). Sampling was non-probabilistic (convenience), recruiting families attending the participating centers.

The sampling technique was non-probabilistic and based on convenience. Recruitment was consecutive within this framework, including all eligible families who attended the participating centers during the study period and met the inclusion criteria. Participants were enrolled from three private pediatric physiotherapy clinics in the provinces of Ourense and Pontevedra (Spain) and from the La Palomera Primary Health Care Center in León (Spain).

### 2.4. Data Collection and Variables

Trained physiotherapists and nurses conducted face-to-face structured interviews with parents/guardians, documenting: sociodemographic data; obstetric and peripartum exposures (intrapartum synOTC, epidural analgesia, mode of delivery, instrumental delivery, Kristeller maneuver); neonatal anthropometrics (birth weight, length, head circumference); gestational age at birth; feeding type (exclusive breastfeeding, formula, mixed) and reasons; parental ages; personal/family histories; maternal substance use. infC was classified per Rome IV.

Data collection adhered to a standardized instrument used uniformly across sites (Supplementary Material). Its content validity was reviewed by an expert panel of clinicians and physiotherapists before data collection. All interviews were conducted face-to-face by trained nurses and physiotherapists who cross-checked parental answers against the information available in pediatric records whenever possible, minimizing recall bias and data entry errors.

To ensure data accuracy, additional verification procedures were implemented. Information on intrapartum synthetic oxytocin exposure, epidural analgesia, and delivery-related characteristics was obtained from hospital discharge reports provided by parents and verified against clinical records when available. In the primary health care center, nurses accessed electronic medical records directly to confirm these data. This procedure ensured that delivery information was based on documented clinical sources rather than parental recall.

Structured interviews were conducted at any time between the infant’s second week and fifth month of life, coinciding with the typical age range for the onset and resolution of infantile colic according to Rome IV criteria. Parents were interviewed during routine visits to primary care nursing or private physiotherapy centers, ensuring ecological recruitment and minimizing recall bias.

Primary exposure was intrapartum administration of synthetic oxytocin (yes/no). Primary outcome was infC during the first five months of life (Rome IV).

Secondary variables included epidural analgesia (yes/no), feeding type, gestational age (weeks), neonatal anthropometrics (including head circumference in cm), sex, mode of delivery, and parental ages.

Mode of delivery was classified as vaginal, instrumental vaginal, or cesarean section (scheduled or unscheduled). Scheduled cesareans were defined as procedures performed without labor or intrapartum oxytocin exposure, whereas unscheduled cesareans followed failed vaginal delivery attempts, often after induction or augmentation with oxytocin or prostaglandins. Scheduled cesareans were retained in the descriptive analysis for transparency of the original cohort but were also analyzed separately as potential confounders. A sensitivity analysis excluding these cases was conducted to confirm the robustness of results.

Labor onset was classified as spontaneous or induced. Synthetic oxytocin exposure referred to either induction or augmentation of uterine activity, as indicated in clinical records.

Interviews and data entry followed a pre-specified codebook. Personal identifiers were not retained in the analytic dataset; data were stored in anonymized files in compliance with European Union General Data Protection Regulation 2016/679 and Spanish Organic Law 3/2018 on the Protection of Personal Data and Guarantee of Digital Rights.

### 2.5. Statistical Analysis

Descriptive statistics included means and standard deviations for continuous variables and counts and percentages (with 95% confidence intervals) for categorical variables. Group comparability was explored using Student’s *t* tests for normally distributed continuous variables and Pearson’s chi-square or Fisher’s exact tests for categorical variables.

To estimate the association between intrapartum synOT exposure and infC, we fitted conditional logistic regression models appropriate for individually matched case–control data and reported adjusted odds ratios (aORs) with 95% CIs. A priori covariates in the main model were infant sex, gestational age (weeks), and birth weight (kg). Secondary analyses evaluated (i) the relation between head circumference and colic and (ii) feeding-type distributions by case status. We also modeled the association between epidural analgesia and synOT administration using logistic regression.

Statistical significance was set at *p* < 0.05. Analyses were performed with IBM SPSS Statistics, version 26.

### 2.6. Ethics

The study complied with the Declaration of Helsinki and applicable data-protection regulations. Written informed consent was obtained from both parents/legal guardians before participation. Ethical approval was granted by the Ethics Committee of the University of León (Spain) (reference: ETICA-ULE-020-2022) and by the Ethics Committee for Research with Medicines of the Health Areas of León and El Bierzo (Spain) (reference: 2264).

## 3. Results

### 3.1. Participants and Sample

**Figure 1:**
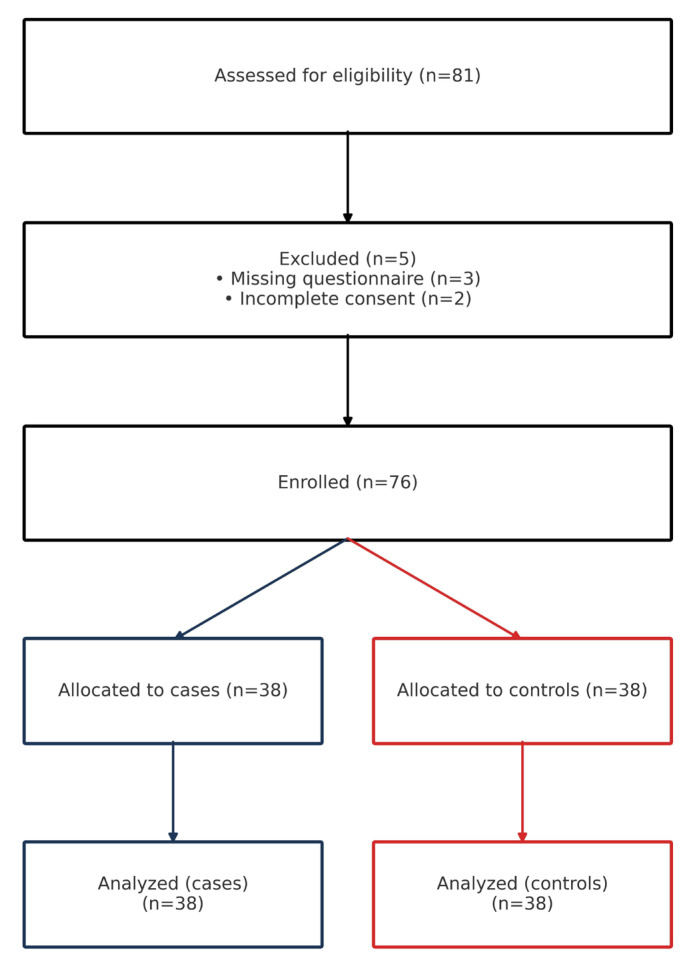
Participant flow diagram.

Between May and June 2022, 81 infant records were screened; 3 were excluded for missing questionnaire data and 2 for incomplete consent, yielding 76 participants (38 cases with infC; 38 controls) (Figure 1). Matching was 1:1 (case–control).

### 3.2. Baseline Characteristics

Groups were broadly comparable in perinatal variables except for head circumference, which was smaller among cases. Sex distribution differed between groups (Table 1). Mean age at colic onset among cases was 22 ± 10.7 days (min 10; max 60).

Among vaginal births, 58.6% were spontaneous, and 41.4% were induced. Oxytocin was used for either induction or augmentation in 51 participants (67.1% of induced labors).

### 3.3. Intrapartum Interventions and Feeding

**Figure 2:**
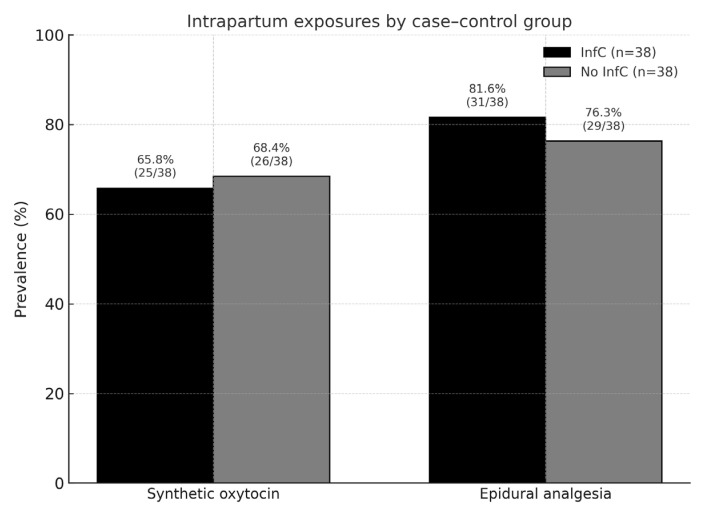
Prevalence of intrapartum exposures in the study sample.

In the overall sample, 57.9% received intrapartum synthetic oxytocin and 81.6% epidural analgesia (Figure 2).

Intrapartum characteristics did not differ significantly between groups (Table 2). SynOTC was used in 66% of InfC and 68% of No InfC deliveries (*p* = 0.81). Epidural analgesia was administered in 82% vs. 76% (*p* = 0.58). Rates of vaginal, instrumental and cesarean delivery were similar between groups. Scheduled cesarean sections represented only 5% in each group and did not affect the association.

At the time of interview, roughly half of infants were exclusively breastfed, and feeding distributions were similar between cases and controls (Table 3).

### 3.4. Primary Association: Synthetic Oxytocin and Infantile Colic

In conditional analyses, synOT showed no association with infC: aOR 1.24 (95% CI 0.50–3.09) (Table 4). The chi-square test was also non-significant (χ^2^ = 0.216; *p* = 0.64).

No significant sex-related differences were found in the distribution of intrapartum oxytocin exposure or in its association with infantile colic.

### 3.5. Secondary Findings

- Epidural and synOT: Epidural use strongly predicted synOT exposure (OR 4.55; 95% CI 1.28–16.20); 2 × 2 counts: epidural yes 40/62 with synOT vs. epidural no 4/14 with synOT; χ^2^ *p* = 0.014 (Table 5).
- Feeding and synOT: No significant association between synOT and reduced breastfeeding (aOR 1.161; 95% CI 0.465–2.900; *p* = 0.749).
- Head circumference: Infants in the InfC group had a smaller mean head circumference at birth compared with controls (33.99 ± 0.96 vs. 34.87 ± 2.32 cm; *p* = 0.035). However, this difference was no longer significant after adjusting for gestational age and other covariates in the multivariable model controls (Table 1).

## 4. Discussion

In this matched case–control study, intrapartum exposure to synOT was not associated with infC during the first five months of life. We also found no independent association between synOT and breastfeeding pattern at follow-up. By contrast, epidural analgesia strongly predicted synOT use, reinforcing the notion that labor analgesia and augmentation are tightly linked in clinical practice.

Our null association between synOT and colic does not preclude short-term neonatal feeding effects reported elsewhere. A recent nationwide study found that higher intrapartum oxytocin dosing correlated with abnormal early sucking patterns and earlier cessation of exclusive breastfeeding, suggesting a dose–response in the immediate postpartum period [12]. Mechanistic work in animals demonstrates transplacental transfer of maternally administered oxytocin and dose-dependent alterations of fetal brain oxytocin receptor expression with sex-specific neurobehavioral changes—biological signals that could plausibly influence neonatal behavior without necessarily translating into a colic phenotype months later [13]. Clinically, however, human data on placental transfer and downstream neonatal effects remain mixed, underlining the need for careful interpretation of observational findings [14].

The biological plausibility of a months-delayed effect from a brief intrapartum oxytocin exposure is uncertain; proposed mechanisms (e.g., short-lived neonatal neurobehavioral changes or epigenetic modulation) remain speculative and not consistently supported in humans. Consistent with this, our data showed no association between intrapartum synthetic oxytocin and infantile colic at 0–5 months.

The microbiome–gut–brain axis offers an alternate explanatory frame for colic risk. Emerging population-based metagenomic analyses indicate that specific early-life microbial signatures (e.g., Akkermansia, Bifidobacterium, Bacteroides profiles) associate with crying/fussing trajectories, consistent with a multifactorial pathophysiology in which perinatal exposures may play, at most, a modest indirect role [15]. In that context, our findings align with a scenario where intrapartum synOT is not a primary driver of colic incidence.

The observed link between epidural analgesia and subsequent synOT use matches contemporary obstetric literature: epidural can slow or dyscoordinate uterine activity, increasing the likelihood of augmentation, and earlier epidural placement has been associated with longer labors and more interventions in some cohorts [15,16]. Beyond individual cases, hospital-level practice variation in oxytocin augmentation is substantial, driven by differing protocols and thresholds for dystocia management [6,7]. Such variation heightens the risk of confounding by indication in studies of synOT and downstream neonatal outcomes—a key methodological caveat for future research.

From a public-health standpoint, our cohort’s high prevalence of synOT (57.9%) and epidural use (81.6%) reflects intervention-dense labor management in the participating sites and underscores the importance of judicious augmentation within guideline frameworks [17]. At the same time, the global prevalence of infC—typically ~10–15% under Rome IV definitions—suggests that even if synOT had small effects, population-attributable impact would likely be limited without large exposure gradients or synergistic factors [2]. Our data neither support a detrimental synOT–colic link nor indicate impaired breastfeeding at 0–5 months; this is consistent with heterogeneous human literature where early breastfeeding difficulties may be seen at high oxytocin doses but longer-term feeding outcomes often converge [12,18].

Our study strengths include prospective 1:1 matching, standardized Rome IV case definition, and trained, in-person interviews minimizing misclassification. Limitations are the modest sample size, potential residual confounding (e.g., unmeasured dosing and timing for synOT; epidural timing), and single-region sampling, which may limit generalizability and reduce power to detect small effects. In addition, information on whether oxytocin was administered for uterine inertia or during the second stage of labor was not systematically recorded, which limits detailed interpretation of intrapartum exposure patterns.

Scheduled cesarean sections, in which no intrapartum oxytocin was administered, were retained in the descriptive analysis for completeness but analyzed separately as a potential confounding factor. Their exclusion in a sensitivity analysis did not materially change the main results. Likewise, the small subgroup of mothers who did not receive epidural analgesia was also retained in the analysis to avoid selection bias. Although their number was limited, excluding them would have artificially restricted the representativeness of the cohort and reduced comparability between groups. Including all exposure categories allowed a more accurate reflection of real-world obstetric practice while confirming that these small subgroups did not influence the direction or significance of the main findings.

We did not quantify oxytocin dose, infusion rate, or duration, precluding dose–response analyses; given evidence that dose matters for early neonatal behaviors [12] and that practice patterns vary widely [6], future studies should incorporate detailed exposure characterization alongside cord blood biomarkers, structured neonatal feeding assessments, and longitudinal microbiome profiling.

The use of a 1:1 matching ratio may have reduced statistical power compared with higher control-to-case ratios; however, this approach ensured strict comparability between groups under the predefined matching criteria.

Our results are reassuring regarding synOT and colic and highlight the need to disentangle epidural–augmentation clusters from causal effects on neonatal outcomes. Pragmatically, aligning augmentation practices with evidence-based labor management guidance and prioritizing breastfeeding support remain appropriate, while research focuses on dose, timing, and susceptibility windows that could modify neurobehavioral or feeding trajectories.

From a clinical and public health perspective, these findings reinforce the need for an integrated approach to childbirth management and postnatal follow-up. Although synthetic oxytocin remains an essential tool for labor induction and augmentation, its use should always be individualized and kept within evidence-based safety limits.

Future studies should incorporate detailed documentation of oxytocin dosing, infusion rate, and duration of exposure, together with hormonal biomarkers and longitudinal microbiome profiling, to clarify the potential neurobiological mechanisms involved in neonatal crying regulation and behavioral outcomes. Prospective multicenter designs with larger sample sizes and standardized oxytocin administration protocols would further strengthen the validity and generalizability of the evidence.

In clinical practice, these results highlight the importance of coordinated work among physicians, nurses, and physiotherapists during the perinatal period. Neonatal physiotherapy, postural education, and breastfeeding support interventions may play a relevant role in preventing or alleviating symptoms associated with infantile colic, while promoting positive bonding and overall family well-being.

## 5. Conclusions

In this case–control study, intrapartum exposure to synthetic oxytocin was not linked to infantile colic. The frequent co-use with epidural seems to reflect routine labor management rather than a causal path to colic. The smaller head circumference seen in cases warrants a closer look using standardized z-scores. Larger studies that capture oxytocin dose, infusion profile, and timing are needed to test mechanisms and clarify any small, context-dependent effects.

## Figures and Tables

**Table 1:** Baseline characteristics by case–control status (*n* = 76).

Variable	InfC (*n* = 38)	No InfC (*n* = 38)	Test/*p*-Value
Female sex, *n* (%)	22 (57.9)	12 (31.6)	χ^2^ *p* = 0.021
Birth weight (kg, mean ± SD)	3.13 ± 0.38	3.22 ± 0.39	t *p* = 0.223
Length at birth (cm, mean ± SD)	48.72 ± 2.88	49.38 ± 1.93	t *p* = 0.245
Head circumference (cm, mean ± SD)	33.99 ± 0.96	34.87 ± 2.32	t *p* = 0.035
Gestational age (weeks, mean ± SD)	39.30 ± 1.36	39.18 ± 1.30	t *p* = 0.680

InfC: Infant Colic (cases); NoInfC; No Infant Colic (controls); t: Student’s t for continuous variables; χ^2^: Pearson’s for categorical variables.

**Table 2:** Intrapartum characteristics and mode of delivery by case–control group (*n* = 76).

Variable	InfC (*n* = 38) *n* (%)	No InfC (*n* = 38) *n* (%)	Test/*p* Value
Synthetic oxytocin	25 (65.8%)	26 (68.4%)	χ^2^ = 0.81
Epidural analgesia	31 (81.6%)	29 (76.3%)	χ^2^ = 0.58
Vaginal delivery	28 (73.7%)	27 (71.1%)	χ^2^ = 0.80
Instrumental delivery	9 (23.7%)	8 (21.1%)	χ^2^ = 0.78
Cesarean (all)	10 (26.3%)	11 (28.9%)	Fisher = 0.81
Scheduled cesarean	2 (5.3%)	2 (5.3%)	Fisher = 1.00

InfC: infantile colic (cases); No InfC: no infantile colic (controls); scheduled cesarean corresponds to cesarean sections with no intrapartum oxytocin exposure; χ^2^: Pearson’s test.

**Table 3:** Feeding type by case–control status (*n* = 76).

Feeding Type	Cases, *n* (%)	Controls, *n* (%)	Total, *n* (%)
Exclusive breastfeeding	20 (52.6)	21 (55.3)	41 (53.9)
Mixed feeding	10 (26.3)	4 (10.5)	14 (18.4)

**Table 4:** Association between intrapartum synthetic oxytocin and infantile colic.

Measure	Estimate (95% CI)	*p*-Value
Adjusted odds ratio (synOT: yes vs. no)	1.24 (0.50–3.09)	—
Pearson’s χ^2^ (2 × 2)	0.216	0.64

synOT: synthetic oxytocin; CI: confidence interval. Model adjusted a priori for infant sex, gestational age (weeks), and birth weight (kg).

**Table 5:** Association between epidural analgesia and synthetic oxytocin.

	synOT Yes	synOT No	Total
Epidural yes	40	22	62
Epidural no	4	10	14
Total	44	32	76

Odds ratio (epidural yes vs. no) = 4.55 (95% CI 1.28–16.20); synOT: synthetic oxytocin.

## Data Availability

The interviewer-administered Spanish questionnaire (including Rome IV items) is available upon request to the corresponding author. Owing to the informed-consent constraints (explicitly restricting data sharing to the study team), the de-identified individual-level dataset cannot be deposited in a public repository. Aggregated tables and the SPSS analysis syntax will be provided upon reasonable request, subject to institutional and ethics approvals.

## Supplementary Materials

The following supporting information can be downloaded at: https://www.mdpi.com/article/10.3390/medicina61111908/s1.

## Abbreviations

The following abbreviations are used in this manuscript:

infC	Infantile Colic
synOTC	Synthetic Oxytocin
