# Supplementary material for: Labor Induction with Synthetic Oxytocin and Infantile Colic: A Case–Control Study
Source: Medicina (Kaunas). 2025 Oct 24;61(11):1908. doi: 10.3390/medicina61111908 (PMC12654489; doi:10.3390/medicina61111908)
Supplement: Supplementary file 1 [file medicina-61-01908-s001.zip › medicina-3904754-supplementary.pdf]

LimeSurvey

Los investigadores agradecemos su colaboración.

Sección A: Consentimiento

A1. **ACEPTO participar voluntaria y anónimamente en la siguiente encuesta. Este estudio es parte de una investigación realizada sobre el cólico del lactante y el uso de oxitocina artificial intraparto. Todas las respuestas son anónimas y estrictamente confidenciales y sólo se utilizarán para fines docentes y de investigación : De acuerdo con el Reglamento Europeo de Protección de Datos (UE) 2016/679 y con la Ley Orgánica 3/2018 de Protección de Datos Personales y Garantía de los Derechos Digitales.**
Agradecemos tu colaboración, quedamos a tu disposición para cualquier duda o aclaración, e informaros de los resultados y conclusiones del estudio si así lo consideráis.
Fdo. Cristina Suárez Fraga (csuarf00@estudiantes.unileon.es)

Sí ☐

No ☐

Sección B: DATOS SOCIODEMOGRÁFICOS Y ANTROPOMÉTRICOS

B1. **Genéro del bebé**

Femenino ☐

Masculino ☐

B2. **Fecha nacimiento bebé**

B3. **Peso al nacer**

B4. **Talla al nacer**

B5. **Perimetro craneal al nacer**

LimeSurvey

B6. **Antecedentes personales**

B7. **Antecedentes familiares (madre , padre, hermanos)**

B8. **Hábitos tóxicos de la madre**

Tabaco ☐
Alcohol ☐
Drogas ☐

B9. **Edad de la madre**

B10. **Edad del padre**

Sección C: DATOS DEL PARTO

C1. **Semana del parto**

C2. **Tipo de parto**

Parto Vaginal ☐
Cesárea ☐
Cesárea Programada ☐
Otro ☐

Otro

C3. **Parto instrumental**

- Sí ☐
- No ☐

C4. **Parto instrumentado**

- Forcéps ☐
- Ventosa ☐

C5. **Epidural**

- Sí ☐
- No ☐

C6. **Oxitocina**

- Sí ☐
- No ☐

C7. **Maniobra de Kristeller**

- Sí ☐
- No ☐

Sección D: ALIMENTACIÓN

D1. **Tipo de alimentación**

- Lactancia materna ☐
- Alimentación artificial ☐
- Alimentación mixta ☐

D2. **Motivo del tipo de alimentación**

Sección E: CÓLICO DEL LACTANTE

E1. **Cólico del lactante**

- Sí ☐
- No ☐

LimeSurvey

E2. **¿Cuando comenzaron los cólicos?**

Ha finalizado la encuesta. Gracias por su colaboración.
